# InterPro (The Integrated Resource of Protein Domains and Functional Sites)

**DOI:** 10.1002/1097-0061(200012)17:4<327::AID-YEA45>3.0.CO;2-K

**Published:** 2000

**Authors:** Christopher Southan

**Affiliations:** Bioinformatics Target Discovery SmithKline Beecham Pharmaceuticals New Frontiers Science Park North Third Avenue, Harlow Essex CM19 5AW UK

## Abstract

The family and motif databases, PROSITE, PRINTS, Pfam and ProDom, have been integrated into a powerful resource for protein secondary annotation. As of June 2000, InterPro had processed 384 572 proteins in SWISS-PROT and TrEMBL. Because the contributing databases have different clustering principles and scoring sensitivities, the combined assignments compliment each other for grouping protein families and delineating domains. The graphic displays of all matches above the scoring thresholds enables judgements to be made on the concordances or differences between the assignments. The website links can be used to analyse novel sequences and for queries across the proteomes of 32 organisms, including the partial human set, by domain and/or protein family. An analysis of selected HtrA/DegQ proteases demonstrates the utility of this website for detailed comparative genomics. Further information on the project can be found at the European Bioinformatics Institute at http://www.ebi.ac.uk/interpro/.


					Website Review
InterPro (The Integrated Resource of
Protein Domains and Functional Sites)
    
Christopher Southan*
   
      
     
     ! 
   "    #
$  %& '() *#+ ,
%-
 .. /0

Received: 15 September 2000
Accepted: 20 September 2000
Abstract
The family and motif databases, PROSITE, PRINTS, Pfam and ProDom, have been
integrated into a powerful resource for protein secondary annotation. As of June 2000,
InterPro had processed 384 572 proteins in SWISS-PROT and TrEMBL. Because the
contributing databases have different clustering principles and scoring sensitivities, the
combined assignments compliment each other for grouping protein families and delineating
domains. The graphic displays of all matches above the scoring thresholds enables
judgements to be made on the concordances or differences between the assignments. The
website links can be used to analyse novel sequences and for queries across the proteomes
of 32 organisms, including the partial human set, by domain and/or protein family. An
analysis of selected HtrA/DegQ proteases demonstrates the utility of this website for
detailed comparative genomics. Further information on the project can be found at the
European Bioinformatics Institute at http://www.ebi.ac.uk/interpro/ Copyright # 2000
John Wiley & Sons, Ltd.
Introduction
           
          
           
     !"##    
       $    % 
%   %     
       % 
  %  %        
       &   
      %    
            
  '   &   
  (  )* et al !"""+,
- )$ . et al /000+,  )1 
et al /000+,  2 )3 et al /000+
  .         % 
           
     &     )4 /000+
   506/       678  
/8!#    89   !8     
 &   )$.  et al /000+   
   %       
 ' %   '    
 .            .    
           
  :1;          
 < )1   $.  /000+
            
  ' 
Site outline
   )=  !+      
2         
             
            
         
       1         
         %   2   
          
    $3;     
   -   <
:1;          
    etc     
  %   )+     > ?
          $     
          
             
          
Yeast
Yeast  17 
  #      !" #$
.  .        %
       :1; 
     $ >      
      <    
 <+:1; )  <$;;+
       
The protein and protein family displays
1       .     
  .         
           % 
       .     
         %   >  %
            
      .    % 
 .       .  
          . 
&           
 .           
       
=  /        
   .        Escher-
ichia coli * $?2@        
      . 3   2A   
  >   &  
  :%  )*:: +   %
Figure 1. % &' (  
328 Website Review
  #      !" #$ Yeast  17 
 B           2A
      .   & 
    .          
- %         >
 &    &   * $?
@             )
C D      +   
C D       *.%    
-  B     . 2A
         % 
    . 2A       
       2A    
 B      
    
=   %         
            
             
 =      - 
           
 2A     .   
      .  .      
    00!87#  .  =  5
   .       & 
            
    <    
 B )        %   
  + 697  90! 2A     %
          
 %  9/"      . 
=  / .     2A
     - C D  
  %          
 %   .   i.e 
         
 
           
      &  >   
   ?  >     
-      =  /    
    & 00!"80     * $?2@
       )=  8+    7"
    &   & 
   - * $?2@   
.    >  2A   
C   D           
00!/68
Figure 2. ) &'    $ !  *  %+,-# .// *(  &'012 3)45  ! * (  http://
www.ebi.ac.uk/servlets/interpro/production/IEntry?ac=IPR001940 % '!  (  !   " '* (   $
'0&6% 
Figure 3. % &' '47 $(   )  &'  !   8! 9 $!   ! $  !   
 $ !  !  !  ! ! !!
Website Review 329
  #      !" #$ Yeast  17 
$     =  6   )   +
  * $         
00!/68      .  
    %  %   
        &
          !55/
      *.%     
     #6/    %   % 
        !!
  >  00!56   
% &    ?)   +  
% +  00!56    "8E .  
<        %
.           % F  
&           
        .    
    &    .  
    %      
   .    59E   &
       % *.% 
  %        %
 00!56   .   >
           . 
     .   50!
    00!56  < 
'  .         
      %   * $?
2@       -   7"  
%  >     00!56  % 
      %  >  
% &           * $?
2@ >         
. .   C D 00!59 
      00!5!8 
Figure 4. % &' 3)45  ! * (  
Figure 5. % &' !  !   ! * (  
330 Website Review
  #      !" #$ Yeast  17 
           &  
   -     
    &        
         )1
et al !""#+
Proteome analysis
       <
   1      %    % 
          
            $
       5!
       
      )http://www.ebi.ac.uk/
proteome/+        
    .  =  9 $   
!#"##    .       
   <  :1;    
           
  . !5 /76 i.e 9"E       
3 % &  G .   
68E 68E  6/E   %    
        ) 
     +     .
.       $  % 
   %    % >  &
  .  %     
       .   
         
     ' %     
>    =  7 .   /0
       .   
           
.    %    %  
    >  = > 
    G .     
5E /E  !#E         % 
           
*.%  %         
Figure 6. )  (   !  !  $ (   9  ! ! *  H. sapiens ( ! !$    !    9
 ! * !   $ 
Website Review 331
  #      !" #$ Yeast  17 
F=      %    
 %         
     1;$    
      
=.       * $?2@ >
  % .          
. 2A          
     .   % % 
    65"/0 i.e         
  2A    &   -  
    ""7     )=  #+ 
    >  . %    % . 
 * $       ) 
< !""7+        %
     65"/0   
         * $?2@
      =  / .  . 2A
           
          $  
           -  
   1;$   67   
   * $?2@       
 000!   3       
   =  #     -
           2A %
  %       
 &     &  
   65"/0    00!/68   
    -    . . 
   . %   
00!"80  * $?2@       
3            
 .       
  .  B $    
       C D  
 65"/0        %
            %
 2A   >     
   65"/0
$       =  7  
Figure 7. % &'    (   ! 9! C. elegans" D. melanogaster $ S. cerevisiae
Figure 8. &' $(  (  ! *  6:(   !   *   !   & :'0;% '.2
332 Website Review
  #      !" #$ Yeast  17 
       2A  ,    
        /9    
   /7 < 80666   
  1;$         
  %
 -     & 
* $?2@       %   
 80 > %       & 
           
           
             
 /00     C. elegans )http://www.ebi.ac.uk/
proteome/CAEEL/interpro/top200.html+  67
2A        !8     
   $         
  % .    %  
% .  2A i.e      
00!"80     .    
 G  /00   )http://www.ebi.ac.uk/
proteome/DROME/interpro/top200.html+  % 96
2A  !"5           
        
00!"80         
           G  
 * $?2@    :1; @"H=45 
     !8/ 2A  !8!   
           
  .      * $?2@
   .   @"/785 85898 
    @"I-6     
  &    .  =  "
3   . =  "  / .
          . 
        %    
         
    2A     . -
     &   
     .       
  B        I 
        00!"80
=00060  =00/!" & &%   
      %  .
        
        
            
   %    %   
           
%           
      % . *.% 
>  % .      
          %  
    .        
   %        
       )http://
www.ebi.ac.uk/Tools/index.html+
Conclusions
        
            
         &    
            
  %       
  %           
             
  %      i.e  
      %     % 
.            
        .   
.   '  .    %
         %
           
   % & >  
           
       ?   
           
      $    
   >        
     %       &
Figure 9. ) &'    $ !  *   (  3)45  ! !<  " %+,-# .//
Website Review 333
  #      !" #$ Yeast  17 
 &  .   &    
 .     
3          
          .  
 '         
        
    .  %   
  )$.      + 
     % .  
:1;  %  .  % 
     %   %% . 
     >    
   .        2
     .       
&          
F   )F+  &   )http://
genome-www.stanford.edu/GO/+  % 
        .     
1  :$ )*  et al /000,  B
et al /000+
) $(!
   '     
1       )1+   <
 F 3 * >  3   IJ
$  % .   .   
.         $.   
    '       . 
 http://www.ebi.ac.uk/interpro/release_notes.html.
Source databases
              
*:: ( http://www.sanger.ac.uk/Software/Pfam/
index.shtml
            
  ( http://www.expasy.ch/prosite/
-    &     ( http://
www.bioinf.man.ac.uk/dbbrowser/PRINTS
          ( http://
www.toulouse.inra.fr/prodom.html
<  :1;( http://www.ebi.ac.uk/
swissprot/Information/information.html
 )   %  +( http://www.ebi.
ac.uk/swissprot/Information/information.html
 1      (     
 .     ( http://www.ebi.
ac.uk/Tools/index.html
    ' )     
 +( http://www.ensembl.org/
References
$.   $ . J 1  $   /000    
              
       Bioinformatics )   +
$ . J 3  :2 =. 2   /000 -
(       .  - Nucleic Acids Res
28( //6//7
1  $ $.   /000  <   
       :1;  /000
Nucleic Acids Res 28( 868#
1  $ 1   2     *. J;
 ;; /000      =   2 
  Nucleic Acids Res 28( /95/99
1 $4 .  -2 <  4= ) + !""# Handbook of
Proteolytic Enzymes $   ( -. K
3 = % = FB 4 J 2 /000 2 
23F(          .
    Nucleic Acids Res 28( /97/9"
*  4F F $   %   *   /000
  %        .   1
2    %  Nucleic Acids Res 28( //#/50
* J 1  = ; 1  $ !""" 
        !""" Nucleic Acids Res 27(
/!6/!"
 :4 < 1< !""7  * $         
Mol Microbiol 26)/+( /0"//!
 B 4 3  2    3 1  /000
:$(  .            
   Nucleic Acids Res 28( /5!/58
334 Website Review
  #      !" #$ Yeast  17 

				
